# Extracellular Matrix Synthesis and Remodeling by Mesenchymal Stromal Cells Is Context-Sensitive

**DOI:** 10.3390/ijms23031758

**Published:** 2022-02-03

**Authors:** Janina Burk, Anna Sassmann, Cornelia Kasper, Ariane Nimptsch, Susanna Schubert

**Affiliations:** 1 Equine Clinic (Surgery, Orthopedics), Justus-Liebig-University Giessen, 35392 Giessen, Germany; 2 Institute for Cell and Tissue Culture Technologies, Department of Biotechnology, University of Natural Resources and Life Sciences (BOKU), 1190 Vienna, Austria; anna.sassmann@stud.fh-campuswien.ac.at (A.S.); cornelia.kasper@boku.ac.at (C.K.); 3 Institute for Medical Physics and Biophysics, University of Leipzig, 04107 Leipzig, Germany; ariane.nimptsch@medizin.uni-leipzig.de; 4 Institute of Human Genetics, University of Leipzig, 04103 Leipzig, Germany; susanna.schubert@medizin.uni-leipzig.de; 5 Saxon Incubator for Clinical Translation, University of Leipzig, 04103 Leipzig, Germany

**Keywords:** mesenchymal stromal cells (MSC), mode of action, extracellular matrix (ECM), MSC-derived ECM, matrisome, collagen, matrix remodeling, matrix metalloproteinases (MMP), tissue-inhibitors-of-matrix-metalloproteinases (TIMP)

## Abstract

Matrix remodeling could be an important mode of action of multipotent mesenchymal stromal cells (MSC) in extracellular matrix (ECM) disease, but knowledge is limited in this respect. As MSC are well-known to adapt their behavior to their environment, we aimed to investigate if their mode of action would change in response to healthy versus pathologically altered ECM. Human MSC-derived ECM was produced under different culture conditions, including standard culture, culture on Matrigel-coated dishes, and stimulation with the pro-fibrotic transforming growth factor-β1 (TGFβ1). The MSC-ECM was decellularized, characterized by histochemistry, and used as MSC culture substrate reflecting different ECM conditions. MSC were cultured on the different ECM substrates or in control conditions for 2 days. Culture on ECM increased the presence of surface molecules with ECM receptor function in the MSC, demonstrating an interaction between MSC and ECM. In MSC cultured on Matrigel-ECM and TGFβ1-ECM, which displayed a fibrosis-like morphology, gene expression of collagens and decorin, as well as total matrix metalloproteinase (MMP) activity in the supernatant were decreased as compared with control conditions. These results demonstrated that MSC adapt to their ECM environment, which may include pathological adaptations that could compromise therapeutic efficacy.

## 1. Introduction

Multipotent mesenchymal stromal cells (MSC) have been attributed a variety of regenerative mechanisms, which are influenced and triggered by external stimuli. Their mode of action in inflammatory or immune-mediated disease is in the focus of many research activities, which have already led to successful therapeutic approaches [1,2]. In contrast, very little is known regarding the possible mechanisms of MSC in extracellular matrix diseases, such as fibrosis.

The extracellular matrix (ECM) is crucial for tissue and organ function. It represents an essential component of the cellular microenvironment, providing not only structure but also and physical and biochemical cues [3,4]. Furthermore, particularly in connective tissues, the ECM is the major contributor to tissue biomechanical properties, with the ECM composition and architecture determining strength and elasticity [5]. The ECM is continuously remodeled by the resident cells, which includes the synthesis of new ECM components and the breakdown of existing ECM components. The ECM turnover relies on the coordinated expression of specific ECM components as well as matrix-degrading enzymes by resident cells, such as matrix metalloproteinases (MMP) and their tissue inhibitors (TIMP) [6,7]. Physiologically, this enables a constant adaptation of the ECM to changing demands, e.g., during development or training and exercise.

However, if the balance between ECM synthesis and breakdown is disrupted, the ECM becomes a key player in pathological processes. In chronic or recurring disease, the persisting attempts of tissue repair are accompanied by the concerted actions of inflammatory mediators and transforming growth factor-β1 (TGFβ1). These recruit and activate collagen-producing (myo)fibroblasts and alter the expression and activation profile of the different MMP and TIMP, eventually leading to fibrosis [8,9,10]. A therapeutic intervention targeting specific pro-fibrotic mediators, for example TGFβ1, is not without difficulty due to their multifaceted actions in diverse other processes [8]. Therefore, multimodal and adaptive therapeutic tools are necessary to treat ECM pathologies; thus, MSC, with their context-sensitive properties, offer promising prospects.

With regard to using MSC to treat ECM pathologies, preclinical studies and phase I/II clinical trials suggested that MSC transplantation could prevent fibrosis in inner organs such as heart, liver, kidney, or lung [11,12,13,14,15]. Yet on the other hand, MSC, or at least their in vivo counterparts, are suspected to drive the development of fibrosis [16]. This discrepancy suggests that both anti- and pro-fibrotic MSC effects may occur and highlights the need to understand the underlying mechanisms in order to prevent misrouted therapeutic attempts.

Part of the anti-fibrotic effects of MSC is the result of modulating inflammatory cascades, but MSC may also directly contribute to matrix remodeling by releasing and activating remodeling enzymes. Yet, so far, there are only few studies specifically addressing this direct matrix-modulatory potential of MSC [17,18,19]. A first study revealed a mutual interplay between MMP activity and MSC behavior, and demonstrated the gene and protein expression of several MMP and TIMP2 by the MSC [19]. Two further studies revealed that MSC inhibit soluble-compartment MMP via secreted TIMP [17] and bind active MMP at their surfaces [18]. This suggests that MSC could actively contribute to selective matrix protection and degradation. Furthermore, mechanical stimulation led to an overall increased gelatinolytic activity [19]. The latter shows that, similar as with other MSC mechanisms, their matrix modulatory activity is altered by external stimuli. Hence, with respect to the question whether MSC will act in an anti- or pro-fibrotic manner, their extracellular environment may be deciding. 

The aim of this study was to investigate if MSC function is affected by ECM produced under fibrotic conditions. Specifically, we aimed to explore how MSC adapt their matrix synthesis and remodeling activities to MSC-derived ECM-based cell culture substrates that mimic physiological and pathological extracellular environments. For this purpose, we used a novel human cell culture model based on MSC-derived ECM, as well as control conditions including a commercially available and frequently used extracellular environment (Matrigel coating). We hypothesized that firstly, the ECM deposited by MSC would reflect different culture conditions and display fibrosis-like characteristics after stimulation with TGFβ1 and that secondly, MSC cultured on these ECM substrates would show different matrix modulatory activities depending on their respective ECM environment.

## 2. Results

### 2.1. MSC-Derived ECM Culture Substrates

**Figure 1:**
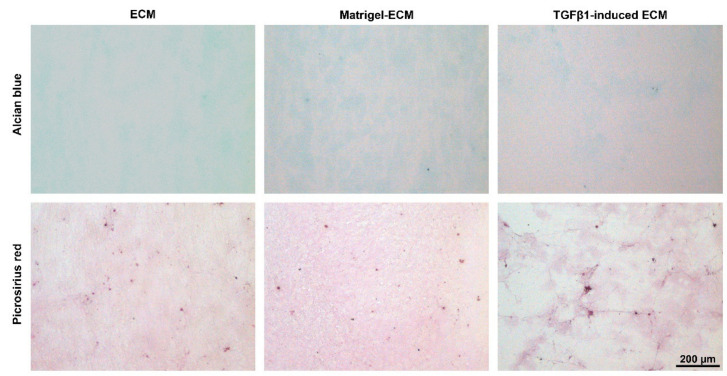
Decellularized MSC-derived ECM obtained after culturing the MSC for 2 days on standard tissue culture plates (ECM), on Matrigel-coated tissue culture plates (Matrigel-ECM), or on standard tissue culture plates with TGFβ1-supplemented medium (TGFβ1-induced ECM). Alcian blue staining was weak, suggesting low glycosaminoglycan content of the MSC-derived ECM, while picrosirius red staining was more distinct, demonstrating the presence of collagens. Note the fibrous structure of the TGFβ1-induced ECM.

After 2 days of culture, the MSC had deposited ECM which could be decellularized and used as culture substrate for the subsequent experiments. Depending on the culture conditions during the time period used to generate the MSC-derived ECM, the structure of the ECM deposited by the MSC was qualitatively different. The ECM obtained after seeding on standard tissue culture plastic (TCP) plates showed a homogeneous structure. In contrast, the TGFβ1-induced ECM, which was obtained after seeding the MSC on TCP plates but with addition of TGFβ1 to the culture medium, had an inhomogeneous morphology with fiber structures. The Matrigel-ECM, obtained after seeding the MSC on Matrigel-coated plates, was distributed less homogeneously than the normal ECM, but lacked the fiber structures of the TGFβ1-induced ECM. Picrosirius staining was positive in all ECM substrates, while Alcian blue staining was rather weak, indicating that the MSC-derived ECM substrates contained more collagens (Figure 1).

### 2.2. Cell Viability, Presence of ECM Receptors and Cytoskeleton Formation

**Figure 2:**
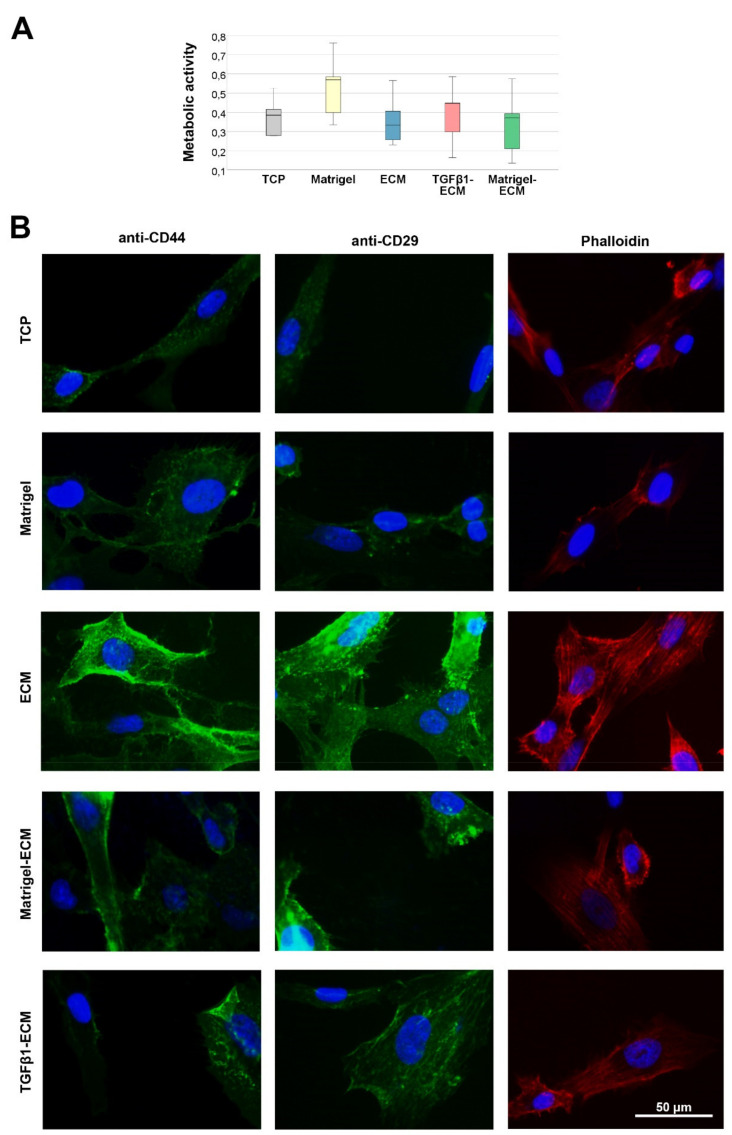
Metabolic activity and microscopy. (**A**) Metabolic activity of MSC after culture on the different MSC-derived ECM substrates (ECM, Matrigel-ECM, TGFβ1-ECM) or in the respective control conditions without MSC-derived ECM (tissue culture plastic (TCP), Matrigel), as determined by MTS-assay (*n* = 5 independent experiments). (**B**) Representative images of surface molecule (CD44, CD29) and actin cytoskeleton (phalloidin) staining of MSC cultured on the different substrates. Note the higher intensity of the stainings in MSC cultured on ECM, indicating that the surface molecules interacting with the ECM are more present and the cytoskeleton formation is more pronounced.

When culturing fresh MSC on the different substrates but without differences in growth factor supplementation, MSC metabolic activity, measured as an indicator for cellular viability, was highest on the Matrigel control substrate. However, it was similar in the TCP control and on all MSC-derived ECM substrates, and differences between groups were overall not significant (Figure 2A).

Staining of CD29 and CD44, both surface molecules interacting with ECM components (integrin-β1 and homing cell adhesion molecule, respectively), demonstrated interaction of MSC and the MSC-derived ECM substrates. Both molecules were more distinctly stained in MSC cultured on any MSC-ECM-based substrate as compared with TCP or Matrigel, and the staining was most abundant on the normal MSC-derived ECM. The same was observed regarding the actin cytoskeleton fiber formation, possibly as a result of activation of the integrin (CD29)/rho/ROCK axis (Figure 2B).

### 2.3. ECM, MMP and TIMP Gene Expression

**Figure 3:**
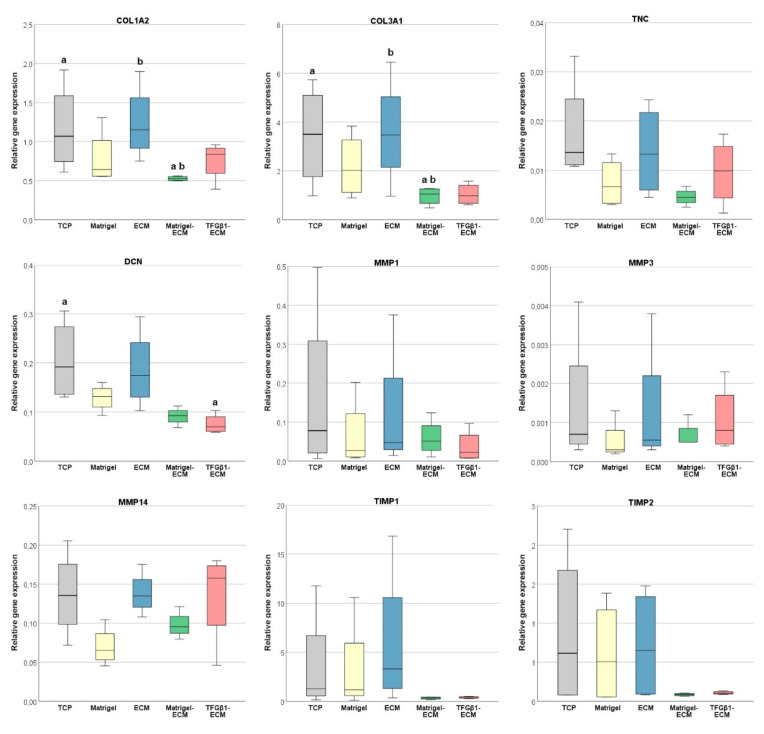
Relative gene expression of the extracellular matrix components collagen 1A2 (*COL1A2*), collagen 3A1 (*COL3A1*), tenascin-C (*TNC*), and decorin (*DCN*), the matrix metalloproteinases *MMP1*, *MMP3*, and *MMP14*, and their tissue inhibitors *TIMP1* and *TIMP2* in MSC after culture on the different MSC-derived ECM substrates (ECM, Matrigel-ECM, TGFβ1-ECM) or in the respective control conditions without MSC-derived ECM (tissue culture plastic (TCP), Matrigel). Gene expression differed significantly between groups designated with the same letter (*p* ≤ 0.007 in the post hoc tests). Data were obtained in *n* = 4 independent experiments.

The expression of genes encoding for selected ECM components was similar in MSC cultured on TCP and in MSC cultured on normal MSC-derived ECM. However, in MSC cultured on Matrigel-ECM, collagen 1A2 (*COL1A2*), collagen 3A1 (*COL3A1*), tenascin-C (*TNC*) and decorin (*DCN*) expression levels were decreased (*p* = 0.007 as compared with TCP and ECM for both *COL1A2* and *COL3A1*). In MSC cultured on TGFβ1-induced ECM, *COL3A1* and *DCN* expression levels were decreased (*p* = 0.002 compared with TCP for decorin) (Figure 3).

*MMP1*, *MMP3*, and *MMP14* were only expressed at relatively low levels in all groups; particularly, *MMP3* expression was very low. The expression of all MMP investigated tended to be at the lowest levels in MSC cultured on Matrigel, but this did not reach significance. Similarly, *TIMP1* and *TIMP2* expression was decreased in MSC cultured on Matrigel-ECM as well as in MSC cultured on TGFβ1-induced ECM, but again this was not significant (Figure 3).

Overall, the relative expression of the most analyzed genes was more consistent between experiments in MSC cultured on Matrigel-ECM and TGFβ1-induced ECM.

### 2.4. MMP Gene Expression and Activity

**Figure 4:**
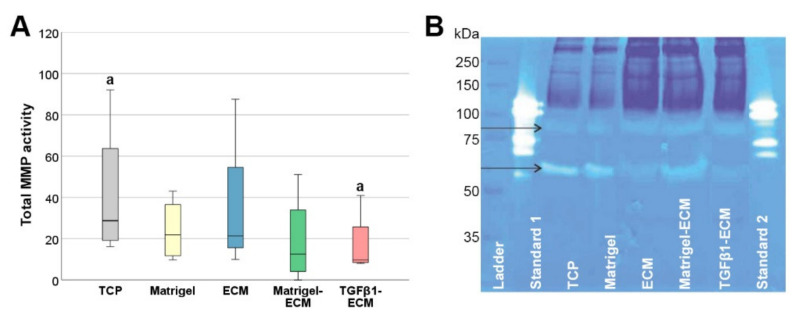
Matrix metalloproteinases (MMP) in cell culture supernatants. (**A**): Total matrix metalloproteinase (MMP) activity, measured in the supernatants of MSC after culture on the different MSC-derived ECM substrates (ECM, Matrigel-ECM, TGFβ1-ECM) or in the respective control conditions without MSC-derived ECM (tissue culture plastic (TCP), Matrigel). MMP activity differed significantly between groups designated with the same letter (*p* ≤ 0.007 in the post hoc tests). (**B**): Representative image of gelatin zymography of the supernatants; bands within the same kDa range were detected in all samples, but with different intensity (arrows); ladder: Precision Plus Protein Dual Color Standards (Bio-Rad, Hercules, CA, USA); standard 1: 0.5 µg collagenase V; standard 2: 1 µg collagenase V.

Although no major differences were found in MMP gene expression, the enzymatic activity of total MMP released into the supernatants was significantly reduced on the TGFβ1-induced ECM substrate (*p* = 0.004 for TGFβ1-induced ECM vs. TCP) (Figure 4A). Zymography revealed that the active MMP found in the supernatants had molecular weights of approximately 80–85 and 60–65 kDa (Figure 4B).

## 3. Discussion

In this study, we showed that MSC are capable of synthesizing ECM that reflects different culture conditions, and that MSC, when cultured on such different ECM substrates, adapt to their environment. This included a reduction of their matrix remodeling activity in the fibrosis-like environment of the TGFβ1-induced ECM, which suggests that maladaptations to pathological environments may occur. While very little is known on the mode of action of MSC in the context of ECM disease so far, this study performed in a human in vitro model contributes to understanding the role of MSC in ECM physiology and pathophysiology.

The synthesis and deposition of ECM by MSC in cell culture has been described and characterized in several previous studies. The core matrisome consists of collagens, glycoproteins, and proteogylcans, while associated molecules such as protease regulators are also present [20,21]. The MSC-derived ECM was relatively consistent across different MSC donors, and also more similar in bone marrow-derived and adipose-derived MSC as compared with the ECM from neonatal dermal fibroblasts [20]. However, it was described that adipose MSC produce more ECM than Wharton’s jelly-derived MSC [22]. It has also been shown that culture conditions impact MSC-derived ECM synthesis, e.g., chemically induced hypoxia increased collagens, TGFβ1, vascular endothelial growth factor, and basic fibroblast growth factor in MSC sheets, which was partly maintained after decellularization [23]. However, the same study showed that particularly TGFβ1 was lost during decellularization [23]. MSC-derived ECM also have been used successfully to improve MSC growth, differentiation, and angiogenic properties [20,24,25,26] or to modulate immune responses [27,28]. In contrast to the current study, however, MSC-derived ECM had not been used to mimic health and disease so far. Yet we considered it as a promising approach to trigger the MSC in a way that would lead to different types of MSC-derived matrices, which could then be used as in vitro model for physiological versus pathologically altered ECM environments.

Aiming to investigate potential alterations of MSC mechanisms while adapting to a diseased extracellular environment, we included TGFβ1-induced MSC-derived ECM in our experimental groups. We hypothesized that the pro-fibrotic factor TGFβ1, added during the culture period used to produce the ECM, would alter the deposited ECM to reflect a fibrosis-like structure, which was confirmed based on the fiber structures observed in the ECM of this group. Furthermore, we assumed that based on the results of a previous study [23], the TGFβ1 added during culture would be removed from the ECM after decellularization, which we could also confirm by negative TGFβ1 immunostaining of the matrices (data not shown). Therefore, we considered the TGBβ1-induced ECM as a suitable cell culture substrate to investigate the influence of fibrotic ECM on MSC. On the other hand, we included MSC-derived ECM synthesized during culture on Matrigel-coated plates in our experimental groups. This was intended to mimic physiological conditions already during MSC-derived ECM synthesis. Yet interestingly and possibly due to the TGFβ contained in the Matrigel, while the structure of the Matrigel-ECM appeared like an intermediate of the normal and the TGFβ1-induced ECM, the results obtained during subsequent cell culture showed several similarities between Matrigel-ECM and TGFβ1-induced ECM. Therefore, the normal ECM, synthesized during standard culture, is to be considered as the most physiological extracellular environment.

MSC culture on the different MSC-derived ECM was feasible and resulted in some distinct differences observed between groups, while MSC viability was similar in the TCP control and the MSC-derived ECM groups. First, we could show that the MSC interacted with the ECM substrates by increasing the presence of the ECM-binding surface molecules CD29 (integrin-β1) and CD44 (homing cell adhesion molecule). This was most evident in the MSC cultures on normal ECM and entailed a distinctly shaped actin cytoskeleton, which is a typical downstream result following integrin activation [29]. Next, we observed differences in the regulation of ECM genes. This involved a downregulation of collagens and decorin in MSC cultured on Matrigel-ECM and TGFβ1-induced ECM. A downregulation of ECM genes in response to the MSC-derived ECM would correspond to previous observations [20]. Yet in the current study, it was not observed in MSC cultured on physiological MSC-derived ECM; thus, this mechanism could be part of the early adaptations of MSC subjected to a pathologically altered ECM environment. Supporting this hypothesis, we observed a similar response with collagen 3A1 downregulation when culturing equine MSC on diseased tendon ECM as compared with healthy tendon ECM in another recent study [30].

Finally, and with the most importance regarding matrix remodeling as a possible MSC mode of action in ECM disease, the activity of MMP in the cell culture supernatant was reduced in the fibrosis-like ECM environment of TGFβ1-induced MSC-derived ECM. To account for possible MMP already present in the MSC-derived matrisome [20], we used supernatants from non-seeded substrates for background correction, so this difference must be due to the MMP released by the MSC during the cell culture experiments. While gene expression and protein activity do not necessarily correspond for various reasons, this still appears surprising against the current MMP and TIMP gene expression findings. Here, there was almost no regulation of the collagenase MMP1 and the stromelysin MMP3, and only an insignificant regulation of the membrane-type MMP14. Furthermore, the TIMP were expressed at lower levels in MSC cultured on Matrigel-ECM and TGFβ1-induced ECM. Yet providing a possible explanation, zymography suggested that the MMP mainly present in the supernatants were neither collagenases nor stromelysins. Rather, the MMP found in the supernatants were in the molecular weight range of gelatinases (such as MMP2 and MMP9) and membrane-type MMP (such as MMP14 and others), with differences between groups observed mainly in the weight range of the membrane-type MMP. This was interesting as recently, it was demonstrated that secreted MMP14 is a suitable predictor for MSC potency to reduce liver fibrosis [31]. Considering this, reduced MMP14 secretion and activation would represent a maladaptation of MSC in fibrotic environment, which may have to be addressed when attempting MSC-based therapies of ECM disease.

It is possible that the observed (mal)adaptation effects only occur during the first days of exposure to pathologically altered ECM. This is even likely as in our recent study on matrix remodeling by MSC in the specific context of tendon disease, we observed the same effect but only during the first week [30]. In this line, it represents a limitation of the current study that only a short period of time (2 days) could be investigated, which was owed to the fact that long-term culture of the MSC on their decellularized, MSC-derived ECM was not feasible with sufficient repeatability. However, even if this adaptation mechanism is transient, it needs to be acknowledged and care must be taken that MSC are not triggered into a pro-fibrotic mode of action.

## 4. Materials and Methods

### 4.1. MSC and Study Design

An immortalized cell line of human adipose-derived MSC (K5 iMSC) [32] was used for all procedures. The cells were cultured in Alpha Minimum Essential Medium Eagle (Life Technologies, Thermo Fisher Scientific, Waltham, MA, USA), supplemented with 2.5% human platelet lysate and 1 U/mL heparin (PL BioScience GmbH, Aachen, Germany), and 0.1% gentamycin (Lonza, Basel, Switzerland), in a humidified atmosphere with 5% CO_2_ and at 37 °C.

Five different culture surfaces were used in the cell culture model to assess matrix modulatory MSC mechanisms: decellularized MSC-derived ECM grown on tissue culture plastic (TCP) (ECM), decellularized MSC-derived ECM grown on Matrigel-coated TCP (Matrigel-ECM), decellularized MSC-derived ECM grown on TCP with TGFβ1 stimulation (TGFβ1-ECM), and Matrigel-coated TCP (Matrigel) and TCP only (TCP) as controls. First, the MSC were used to produce the respective MSC-derived decellularized ECM culture substrates. In the second step, the MSC were seeded and cultured on these different ECM and control surfaces, after which the MSC and their supernatants and ECM were analyzed for possible differences in matrix modulatory activity and ECM composition, respectively.

### 4.2. Preparation and Evaluation of Cell Culture Substrates

First, part of the TCP dishes were coated with Matrigel (BD Matrigel™ Basement Membrane Matrix; BD BioSciences, Bedford, MA, USA) diluted 1:50 in standard culture medium and working on ice, according to the manufacturer’s instructions. Next, to prepare the MSC-derived ECM, MSC were seeded in standard culture medium at a density of 3000 cells/cm^2^ either on the Matrigel-coated dishes or on TCP dishes. TGFβ1 stimulation was performed by adding 10 ng/mL recombinant human TGFβ1 (catalogue number 240-B; R&D Systems, Minneapolis, MN, USA) to the culture medium. After 3 days, all MSC cultures were decellularized by rinsing the dishes with 0.1 M NH_4_OH and 0.5% Triton X-100 (Sigma Aldrich, St. Louis, MO, USA) in phosphate-buffered saline (PBS) for 3 s. The dishes with the resulting MSC-derived ECM were then left open to dry for 1 h at room temperature, rinsed with PBS, dried again for 1 h, and covered and stored overnight at 4 °C until being used in the cell culture experiments.

Additional dishes equally prepared with ECM substrates were fixed with 4% paraformaldehyde and analyzed by histochemical staining and light microscopy. Alcian blue staining was used to evaluate glycosaminoglycans and picrosirius red staining to evaluate collagen deposition.

### 4.3. MSC Culture Experiments

MSC were seeded on the different surfaces (TCP, Matrigel, ECM, Matrigel-ECM, TGFβ1-ECM) at a density of 6000 cells/cm^2^ and incubated at standard culture conditions as described above. Non-seeded cell culture substrates were incubated accordingly. After 2 days, MSC were analyzed regarding cell viability, fixed for (immuno)fluorescent stainings, and harvested for qRT-PCR gene expression analysis. Furthermore, the supernatants were collected for MMP activity analysis and zymography. Independent experiments were reproduced on different days (at least *n* = 4).

### 4.4. Cell Viability Assay

The viability of the cells cultured on the different substrates was measured using the colorimetric CellTiter 96^®^ AQueous One Solution Cell Proliferation Assay (Promega, Mannheim, Germany). The assay procedure was performed as recommended by the manufacturer, and absorption was measured at 490 nm using an Infinite M1000 plate reader (Tecan, Maennedorf, Switzerland). Background correction was performed based on the absorbance values from corresponding non-seeded substrates that had been incubated accordingly.

### 4.5. (Immuno-)Fluorescence Staining

The cell surface molecules CD29 (integrin-β1) and CD44 (homing cell adhesion molecule) as well as the actin cytoskeleton were stained for fluorescence microscopy. For CD44 and phalloidin staining of the actin fibers, samples were fixed in 4% paraformaldehyde; for CD29 staining, they were fixed in methanol. Unspecific binding sites were blocked with 10% fetal bovine serum for 30 min and primary antibodies (mouse anti-human integrin-β1 antibody (clone P5D2, diluted 1:100; Abcam, Cambridge, UK) and rat anti-mouse/human CD44 antibody (clone IM7, diluted 1:100; Biolegend, San Diego, CA, USA) were then added for 1 h at room temperature. Samples were washed with PBS three times and blocked with 5% rabbit serum for 30 min before incubation with the secondary antibodies (Alexa Fluor™ 488 F(ab’)2 fragment of rabbit anti-mouse IgG (H + L) or Alexa Fluor™ 488 rabbit anti-rat IgG (H + L) (diluted 1:100; Invitrogen, ThermoFisher Scientific, Waltham, MA, USA) for 30 min at room temperature. Tetramethylrhodamine (TRITC) phalloidin (Invitrogen, ThermoFisher Scientific) staining was performed at a concentration of 200 units/mL for 30 min. All samples were counterstained with DAPI (4′,6-Diamidine-2′-phenylindole dihydrochloride (Sigma-Aldrich), diluted 1:1000, for 10 min. Microscopy was performed using a Leica DM IL LED microscope and the imaging software LAS X V4.6 (Leica Microsystems, Wetzlar, Germany).

### 4.6. Real Time RT-PCR

The relative expression of genes encoding for matrix-modulating enzymes, *MMP1*, *MMP3*, *MMP14*, *TIMP1* and *TIMP2*, and for matrix components (collagen 1A2 (*COL1A2*), collagen 3A1 (*COL3A1*), decorin (*DCN*), and tenascin-C (*TNC*) were analyzed by real time RT-PCR. Briefly, mRNA was isolated using the RNeasy^®^ Mini Kit with on-column DNase digestion (Qiagen, Hilden, Germany). RNA was converted to first strand cDNA using Reverse Transcriptase RevertAid H Minus (ThermoFisher Scientific). Next, cDNA was mixed with the respective primers (Table 1) and iQ™ SYBR Green Supermix (Bio-Rad Laboratories, Hercules, CA, USA). Relative quantification of cDNA was performed using an Applied Biosystems™ 7500 Real Time PCR System, and gene expression ratios were calculated according to the Pfaffl method.

### 4.7. MMP Activity Assay and Zymography

The supernatants were analyzed using a fluorometric total MMP activity assay (catalogue number ab112146; Abcam, Cambridge, UK). The procedures were performed as recommended by the manufacturer, with 3 h incubation time for MMP activation, thereby focusing on the collagenase MMP1. Fluorescence was measured at Ex/Em 490/525 nm on the Tecan Infinite M1000 plate reader, and data from corresponding samples incubated without MSC were used for background correction.

To further analyze the MMP, supernatant samples from 4 independent replicates were lyophilized and subjected to zymography. The running gel was prepared with 375 mM Tris–Cl (pH 8.8), 10% acrylamide/bis-acrylamide, 0.1% gelatin, 0.1% SDS, 0.1% ammonium persulphate, and 0.05% TEMED. The stacking gel was composed of 125 mM Tris–Cl (pH 6.8), 3% acrylamide/bis-acrylamide, 0.1% SDS, 0.1% ammonium persulphate, and 0.05% TEMED. Samples were dissolved in Laemmli buffer under non-reducing conditions and electrophoresis was performed in a Tris–glycine buffer (25 mM Tris, 192 mM glycine, 0.1% SDS) at 150 V for 75 min. The gels were washed in washing buffer (2.5% Triton-X, 50 mM Tris-HCl (pH 7.5), 5 mM CaCl_2_, 1 µM ZnCl_2_) twice for 30 min, then they were incubated in digestion buffer (1% Triton-X, 50 mM Tris-HCl (pH 7.5), 5 mM CaCl_2_, 1 µM ZnCl_2_) for an initial 10 min and after repeated buffer feed for 24 h at room temperature, at slow movement on a rocker. Staining was performed for at least 30 min with a staining solution composed of 0.02% coomassie brilliant blue (CBB-G250), 5% Al_2_(SO_4_)_3_, 10% Ethanol, and 2% H_3_PO_4_ (85%). Reagents were purchased from Sigma Aldrich at the highest available purities.

### 4.8. Statistical Analysis

Mean values from technical replicates were calculated for each independent experiment. Statistical analysis was performed with SPSS Statistics 28 software (IBM, New York, NY, USA). First, differences between all groups were analyzed using Friedman tests. In case of significance (*p* ≤ 0.05), pairwise post hoc tests were included and the *p* value considered as significant was Bonferroni-corrected based on the number of relevant comparisons (TCP vs. all groups, Matrigel vs. Matrigel-ECM, ECM vs. Matrigel-ECM and TGFβ1-ECM) (*p* ≤ 0.007).

## 5. Conclusions

We here present the first study on matrix remodeling as potential MSC mode of action in ECM disease, using a novel human in vitro model for physiological and pathologically altered ECM. Our data showed that as hypothesized, the matrix modulatory behavior of MSC depends on the condition of their respective ECM environment, and that the activity of MMP released by the MSC is reduced in fibrosis-like conditions. Our data therefore suggested that maladaptations of the MSC have to be expected when MSC are directly transplanted into fibrotic tissues or organs, which could decrease the therapeutic efficacy or even reverse the anticipated beneficial effects of MSC.

## Figures and Tables

**Table 1:** Primers used for real time RT-PCR.

Gene	Primer Sequences	Accession Number	PCR Product
*GAPDH*	CCACTCCTCCACCTTTGACG CCCTGTTGCTGTAGCCAAATTC	NM_002046.7	101 bp
*COL1A2*	CAAGGACAAGAAACACGTCTGG CAAGGACAAGAAACACGTCTGG	NM_000089.4	101 bp
*COL3A1*	TGAATATCGAACACGCAAGGC AAAGCAAACAGGGCCAACG	NM_000090.4	109 bp
*DCN*	GGAGATACAGCCATCCACCTTC CCAGGTTATAAAAATGAGGGCTTTC	NM_001920.5	102 bp
*TNC*	CTCTGGAAGACACCGTTGGC GAAGTGGTGTTTCTTGGAAGCTG	NM_002160.4	101 bp
*MMP1*	CACAGCTTCCCAGCGACTCT TTCAGCATCTGGTTTCCCAGT	NM_002421.4	195 bp
*MMP3*	CCCGAGGTTGGACCTACAAG ATAGGCTGAGCAAACTGCCA	NM_002422.4	132 bp
*MMP14*	GGCGGGTGAGGAATAACCAA ACGCCTCATCAAACACCCAA	NM_004995.4	156 bp
*TIMP1*	GCTGGAAAACTGCAGGATGG TCCGTCCACAAGCAATGAGT	NM_003254.3	191 bp
*TIMP2*	TCCAAAGCCACCTTAGCCTG GTACAGCATGAAAACGCCCG	NM_003255.5	196 bp

## Data Availability

The data presented in this study are available on request from the corresponding author.
